# Supplementary figures and images for: Folliculin variants linked to Birt-Hogg-Dubé syndrome are targeted for proteasomal degradation
Source: PLoS Genet. 2020 Nov 2;16(11):e1009187. doi: 10.1371/journal.pgen.1009187 (PMC7660926; doi:10.1371/journal.pgen.1009187)

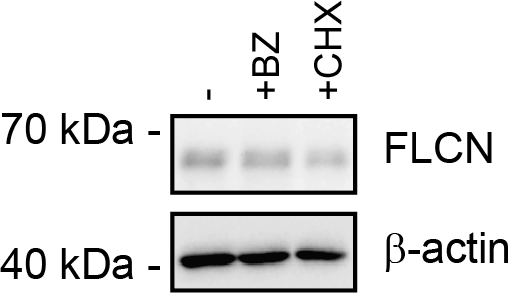

Supplement: S1 Fig — The level of endogenous FLCN in U2OS cells was analyzed by Western blotting of whole cell lysates, using antibodies to FLCN, in cultures that were either untreated (-) or treated with bortezomib (+BZ) or cycloheximide (+CHX) for 8 hours. β-actin served as a loading control. (JPG) [file pgen.1009187.s001.jpg]

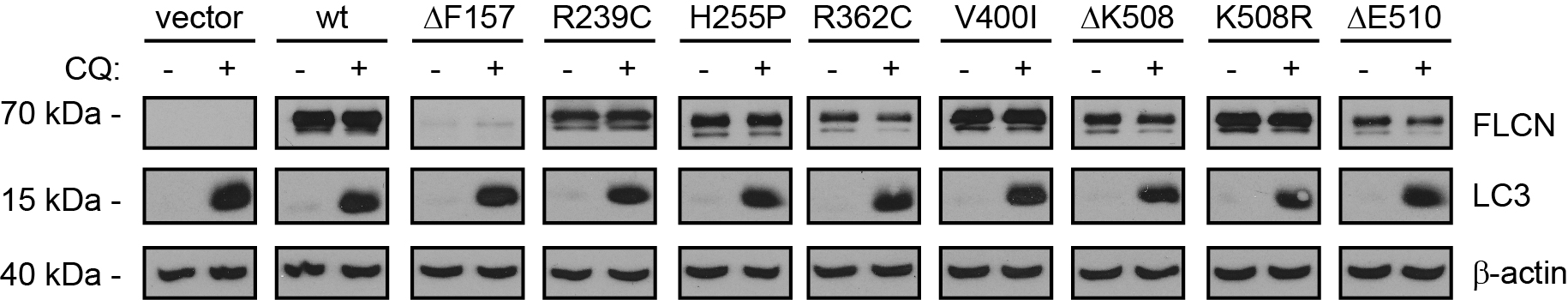

Supplement: S2 Fig — The steady-state levels of the FLCN variants were compared by Western blotting of whole cell lysates, using antibodies to FLCN, in cultures that were either untreated or treated with the autophagy inhibitor chloroquine (CQ) for 8 hours. β-actin served as a loading control, while blotting for the autophagy substrate, LC3, was included as a control for successful inhibition of autophagy. (JPG) [file pgen.1009187.s002.jpg]

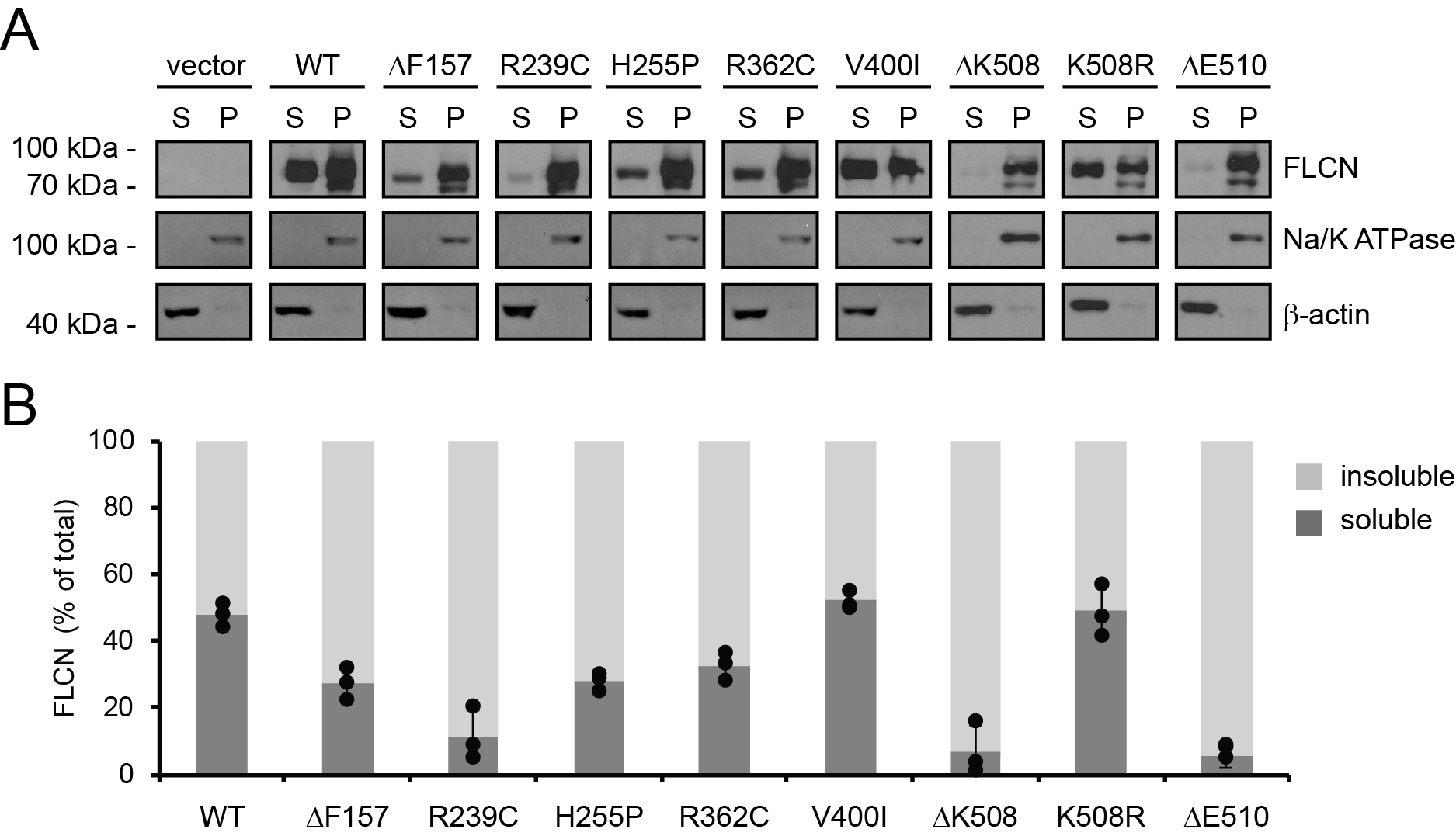

Supplement: S3 Fig — (A) The solubility of the selected FLCN variants. Samples of whole cell lysates were separated into a soluble supernatant (S) fraction and an insoluble pellet (P) fraction by centrifugation. FLCN concentrations were determined by SDS-PAGE and Western blotting with antibodies to FLCN. Na/K ATPase and β-actin were used as loading controls. (B) Quantification of blots as shown in (A) by densitometry. The soluble fractions are shown in dark grey, the insoluble pellet fractions in light grey. For quantification the faster migrating band was included. The error bars show the standard deviation (n = 3). (JPG) [file pgen.1009187.s003.jpg]

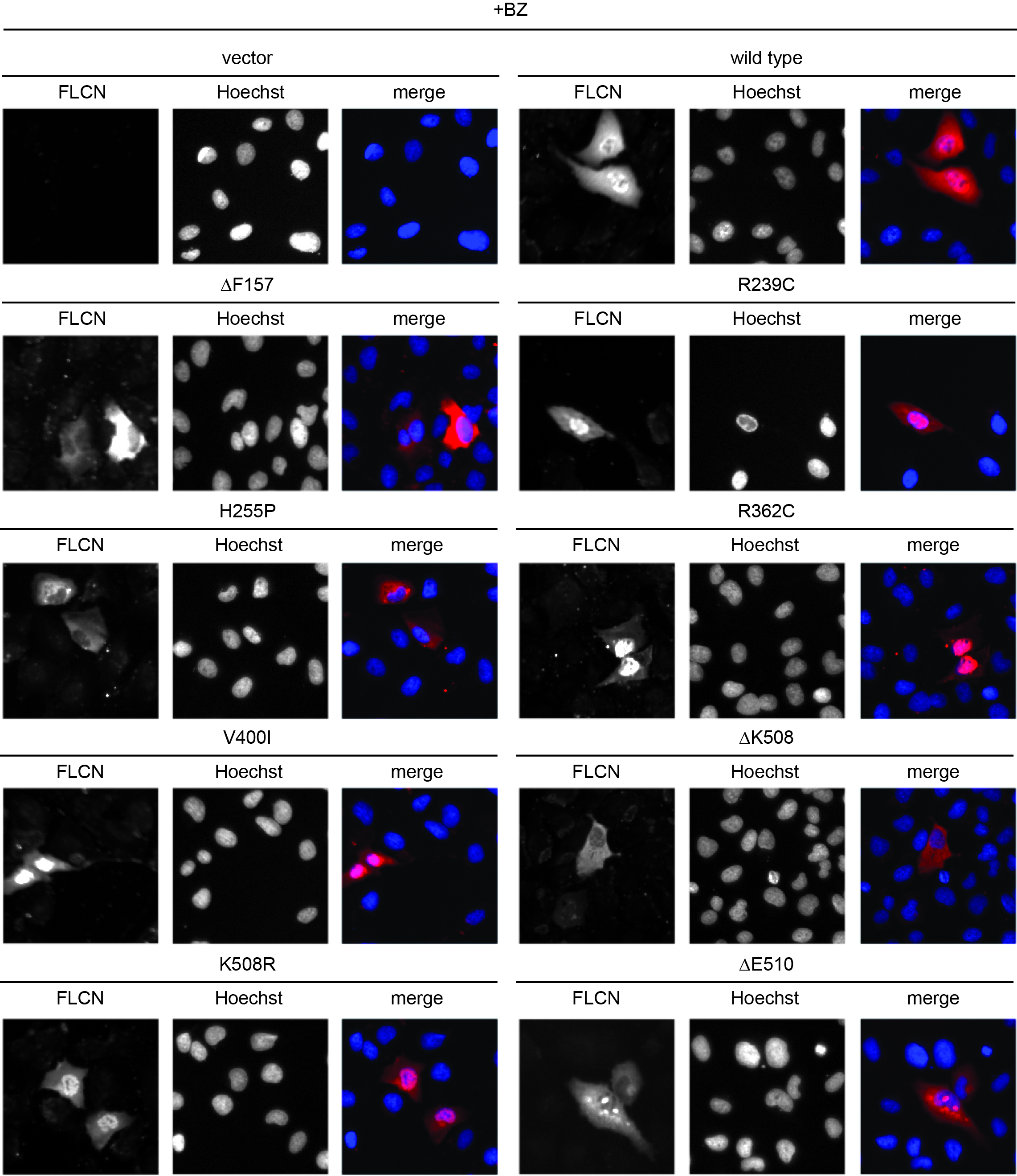

Supplement: S4 Fig — U2OS cells transiently transfected to express 6His-tagged wild type FLCN and selected FLCN variants were treated with the proteasome inhibitor bortezomib (BZ) for 8 hours and analyzed by fluorescence microscopy. FLCN was stained using antibodies to the 6His-tag, and Hoechst was used to mark the nucleus. (JPG) [file pgen.1009187.s004.jpg]

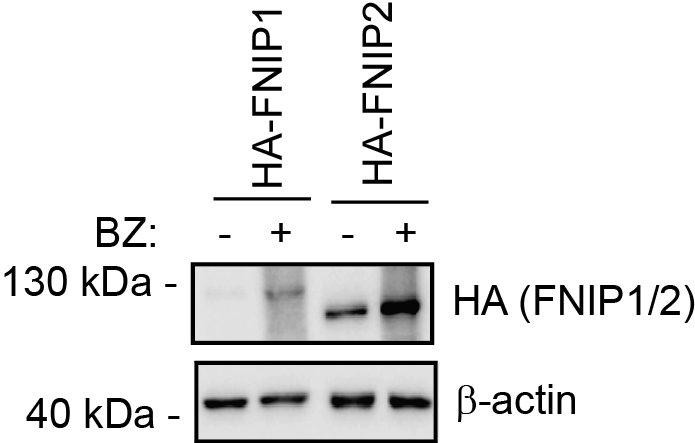

Supplement: S5 Fig — The level of overexpressed HA-tagged FNIP1 and FNIP2 in U2OS cells was analyzed by Western blotting of whole cell lysates, using antibodies to the HA-tag, in cultures that were either left untreated (-) or treated (+) with bortezomib (BZ) for 8 hours. β-actin served as a loading control. (JPG) [file pgen.1009187.s005.jpg]

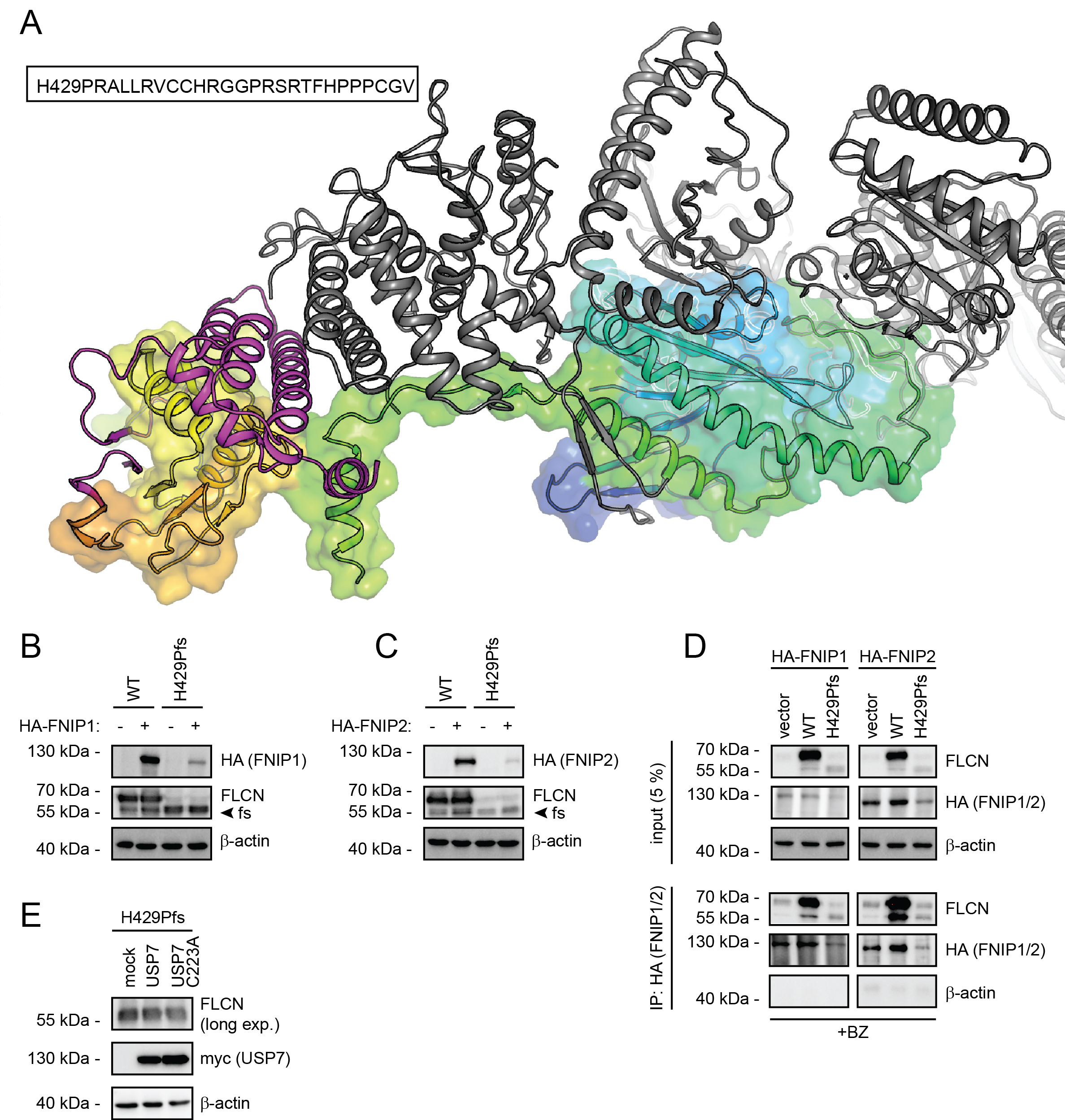

Supplement: S6 Fig — (A) The FLCN H429Pfs variant leads to addition of the shown residues before terminating (boxed sequence). The structure of the FLCN-FNIP2 complex is shown based on the recently resolved cryo-EM structure of FLCN (coloured) and FNIP2 (gray) (PDB 6ULG) (Shen et al., 2019). The purple region is missing in the H429Pfs variant. (B) U2OS cells were transiently transfected to express either wt FLCN or H429Pfs, with or without expression of HA-tagged FNIP1 as indicated. Then the levels of FLCN and FNIP1 in whole cell lysates were compared by SDS-PAGE and Western blotting with antibodies to FLCN or the HA-tag on FNIP1. β-actin served as a loading control. (C) U2OS cells were transiently transfected to express either wt FLCN or H429Pfs, with or without expression of HA-tagged FNIP2 as indicated. Then the levels of FLCN and FNIP2 in whole cell lysates were compared by SDS-PAGE and Western blotting with antibodies to FLCN or the HA-tag on FNIP2. β-actin served as a loading control. (D) U2OS cells were transiently transfected to express either vector, wt FLCN or H429Pfs, and HA-tagged FNIP1 or FNIP2 as indicated, and treated with bortezomib (+BZ) for 8 hours. Cleared extracts (input) were prepared and used for immunoprecipitation (IP) using antibodies to the HA-tag on FNIP1/2. Finally, the samples were resolved by SDS-PAGE and analyzed by Western blotting with antibodies to FLCN or the HA-tag on FNIP1/2, and as a control to β-actin. (E) The level of transfected H429Pfs in U2OS was compared upon co-transfection with myc-tagged USP7 and the catalytically dead USP7 variant (C223A) by Western blotting of whole cell lysates. FLCN was detected using the antibody to FLCN. USP7 was detected by using antibodies to the myc-tag. Probing for β-actin was included as a control. (JPG) [file pgen.1009187.s006.jpg]

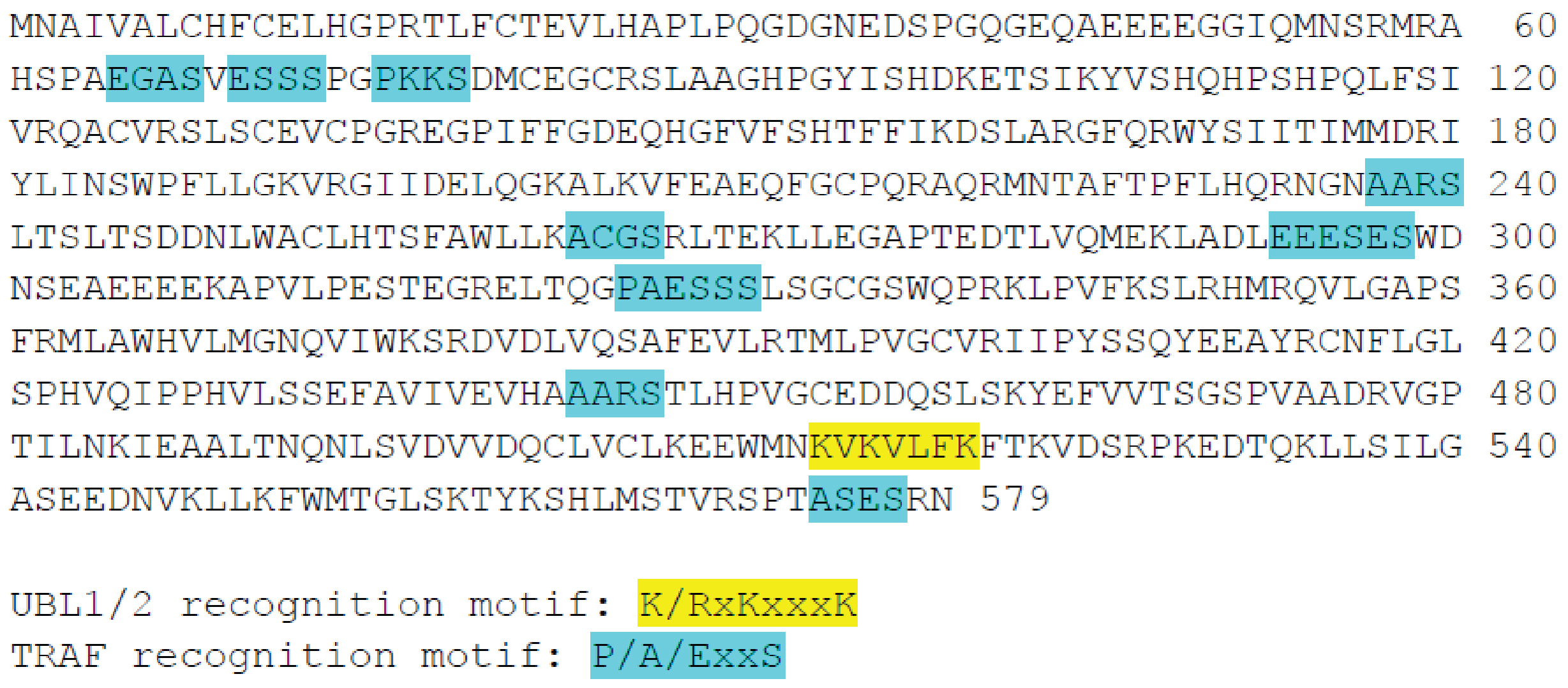

Supplement: S7 Fig — The figure depicts the amino acid sequence of FLCN with the USP7 UBL1/2 (yellow) and TRAF recognition motifs (cyan) marked. The consensus sequences as defined by Kim and Sixma (Kim and Sixma, 2017) of the recognition motifs is given below. The x denotes any amino acid residue. (JPG) [file pgen.1009187.s007.jpg]

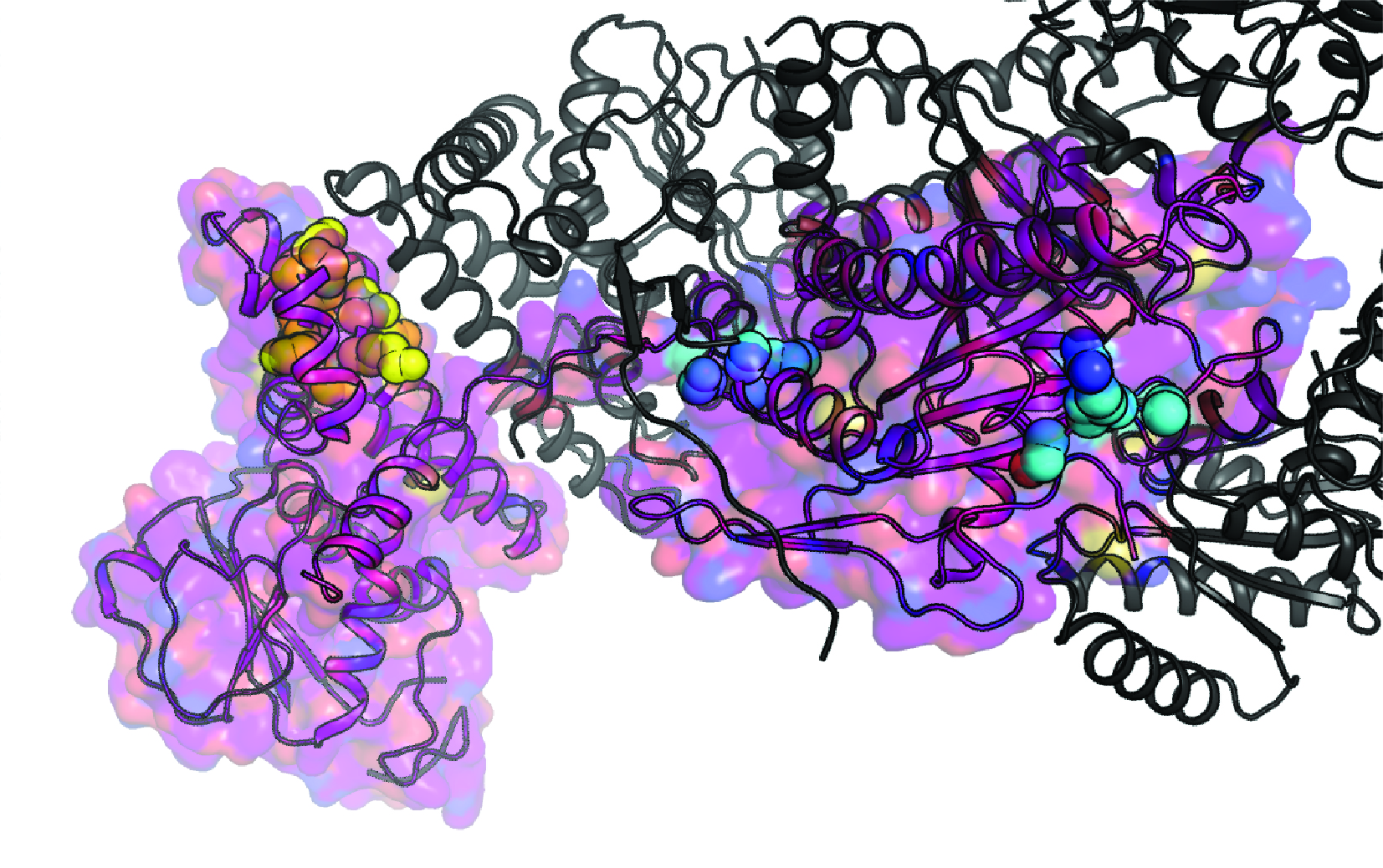

Supplement: S8 Fig — Mapping of the putative USP7 binding sites to the recently resolved cryo-EM structure of FLCN (PDB 6ULG) (Shen et al., 2019) with FLCN in magenta and FNIP2 in gray. Only 3 of the 10 putative sites correspond to regions that are resolved in the structure, namely 236AARS, 264ACGS, which are both in the N-terminal domain, and the UBL1/2 recognition motif (KVKVLFK) in the C-terminal domain. Two of these sites are covered by interaction interfaces with FNIP2 and are thus likely not accessible unless the complexes dissociates. Color coding of motifs as in S7 Fig. (JPG) [file pgen.1009187.s008.jpg]

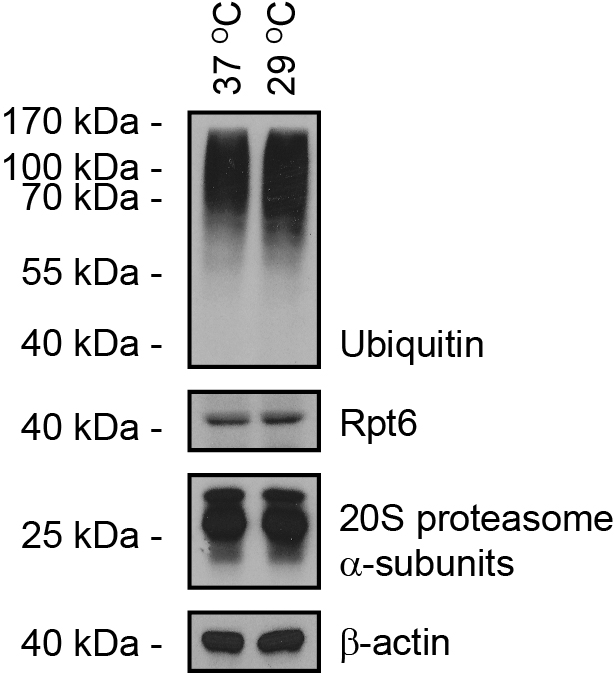

Supplement: S9 Fig — The levels of ubiquitin-protein conjugates and 26S proteasomes in whole cell lysates was compared between U2OS cells grown at 29°C and at 37°C by blotting for ubiquitin, the 19S regulatory complex subunit Rpt6 and the 20S proteasome α-subunits. β-actin served as a loading control. (JPG) [file pgen.1009187.s009.jpg]

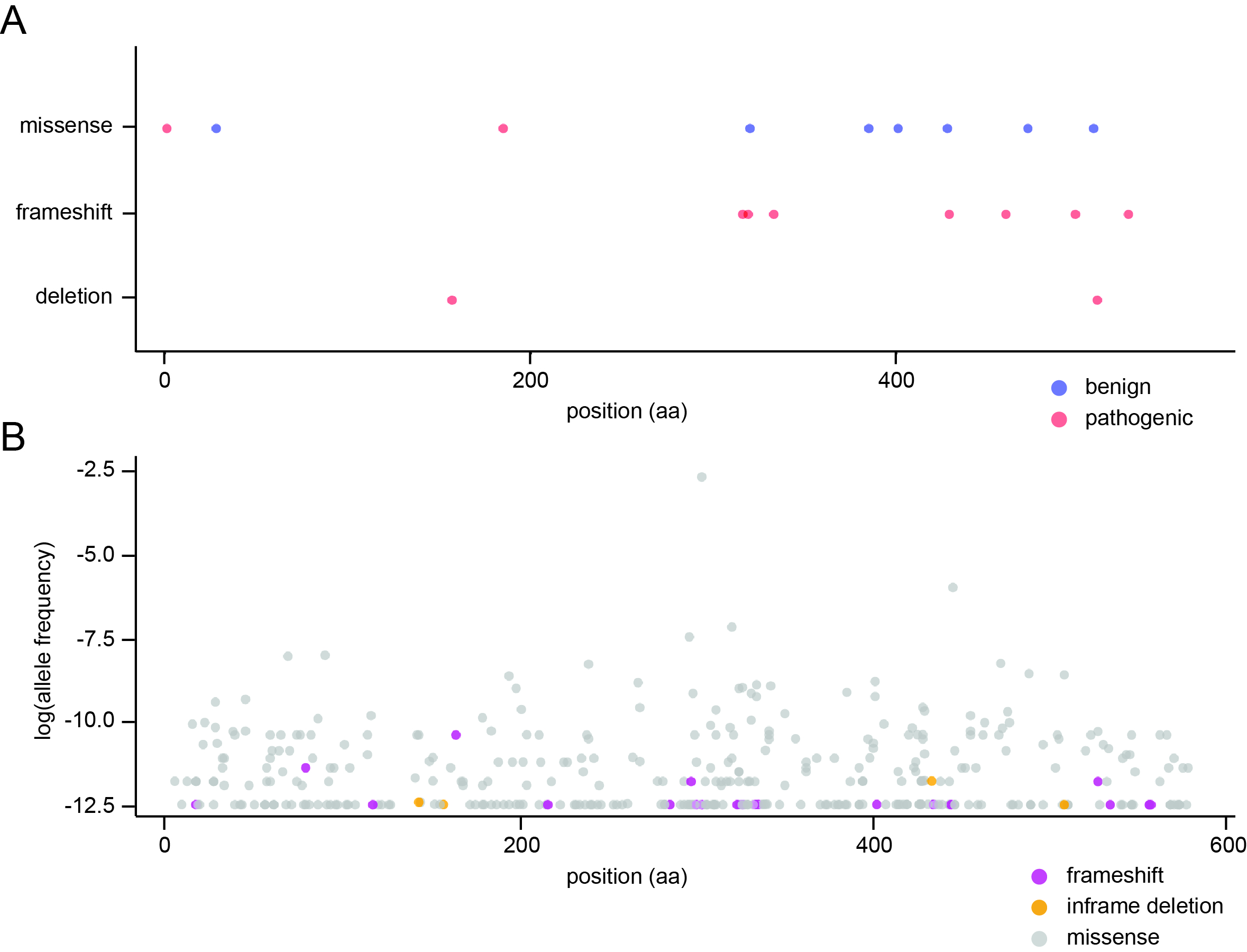

Supplement: S10 Fig — (A) Distribution of missense, frameshift and deletion variants in FLCN coding regions listed in ClinVar. Blue, benign. Red, pathogenic. (B) Allele frequency and distribution of the FLCN variants reported in gnomAD. Purple, frameshift. Yellow, inframe deletion. Grey, missense. (JPG) [file pgen.1009187.s010.jpg]

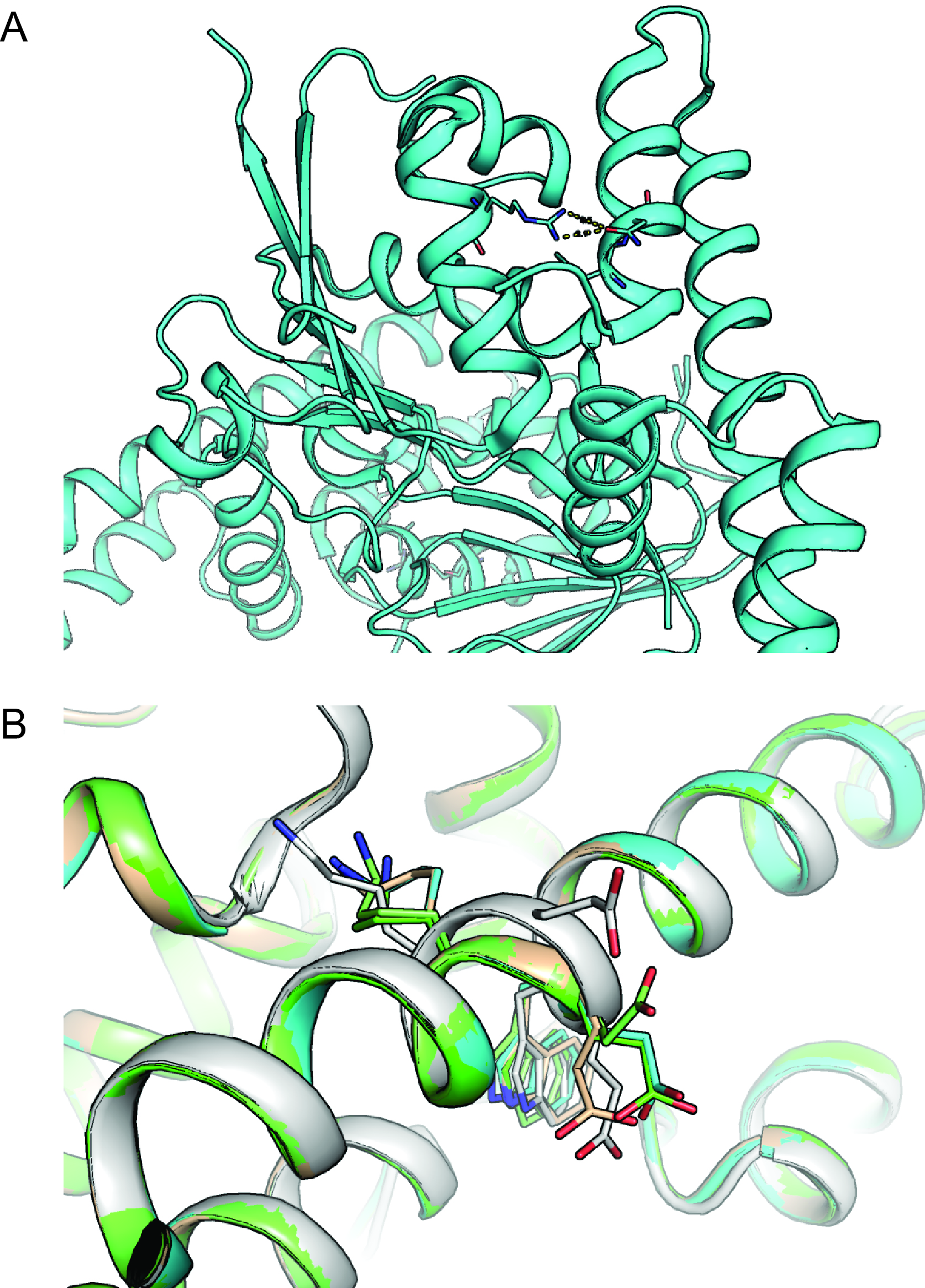

Supplement: S11 Fig — (A) The FLCN R362 residue forms H-bonds with the sidechain of N484 and possibly the backbone carbonyl-O of L483. Accordingly, R362C substitution is expected to be destabilizing, since a C at this position will be unable to engage in these interactions. (B) Introducing a deletion at position E510 is likely to distort the following part of the helix. The models are based on the FLCN crystal structure (PDB: 3V42). (JPG) [file pgen.1009187.s011.jpg]
